# Association between the antioxidant properties of SESN proteins and anti-cancer therapies

**DOI:** 10.1007/s00726-023-03281-6

**Published:** 2023-06-07

**Authors:** Joanna Kozak, Katarzyna Jonak

**Affiliations:** 1 Chair of Fundamental Sciences, Department of Human Anatomy, Medical University of Lublin, Kazimierza Jaczewskiego 4, 20-090 Lublin, Poland; 2 Department of Foreign Languages, Interfaculty Centre for Didactics, Medical University of Lublin, 20-081 Lublin, Poland

**Keywords:** SESN proteins, Oxidative stress, ROS, Autophagy, Anti-cancer therapies

## Abstract

Since the beginning of SESN protein development, they have attracted highly progressive attention due to their regulatory role in multiple signalling pathways. Through their antioxidant activity and autophagy regulation implication, they can function as powerful antioxidants to reduce oxidative stress in cells. SESN proteins received special attention in the field of regulation of reactive oxygen species level in the cell and its interplay with signalling pathways determining energy and nutrient homeostasis. Since perturbations in these pathways are implicated in cancer onset and development, SESNs might constitute potential novel therapeutic targets of broad interest. In this review, we discuss the impact of SESN proteins on anti-cancer therapy based on naturally occurring compounds and conventionally used drugs that influence oxidative stress and autophagy-induced cellular signalling pathways. The significant changes in reactive oxygen species level and nutrient status in cancer cells generate subsequent biological effect through the regulation of SESN-dependent pathways. Thus, SESN may serve as the key molecule for regulating anti-cancer drugs’ induced cellular response.

## Introduction

**Fig. 1:**
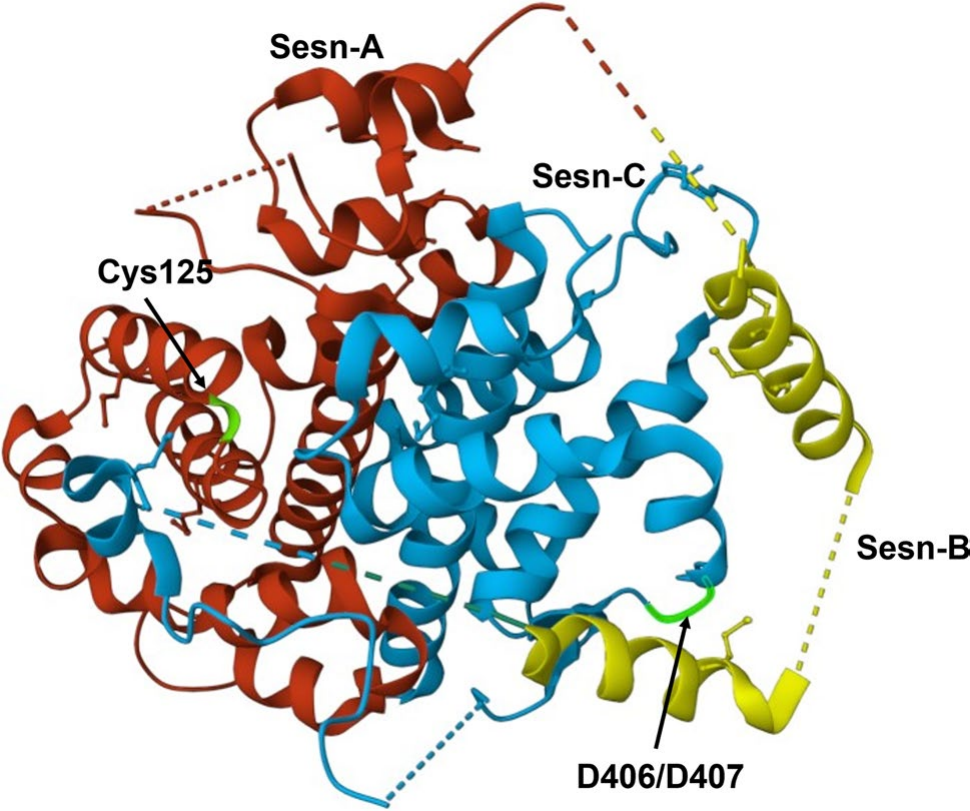
Crystal structure of human SESN2 protein. Sesn-A, Sesn-B and Sesn-C domains are in red, yellow and blue, respectively. Key residues (C125, D406 and D407) in each of the globular domains are displayed in green and indicated by black arrows. Illustrations of the protein structure used in the figure were generated with RCSB Protein Data Bank (RCSB PDB)

Sestrin (SESN) protein family is composed of three distinct members: SESN1, SESN2 and SESN3. These highly conserved proteins are encoded by genes whose expression is regulated by various conditions including hypoxia, oxidative stress, metabolic stress, or DNA damage (Budanov et al. 2010; Lee et al. 2013). The very first pieces of information about the firstly discovered SESN1 (initially known as PA26) came from experiments with p53 response genes under hypoxia, radiation and chemotherapeutic agents’ conditions (Velasco-Miguel et al. 1999). SESN2 (initially known as Hi95) was discovered as a novel gene participating in cellular response to prolonged hypoxia (Budanov et al. 2002). The third member of SESN protein family was discovered by an in silico analysis (Budanov et al. 2002; Peeters et al. 2003). Although SESN proteins show a high percentage of sequence homology, the corresponding genes are located in different human chromosomes, SESN1 on 6p21, SESN2 on 1p35.3 and SESN3 on 11q21 (Velasco-Miguel et al. 1999; Budanov et al. 2002; Peeters et al. 2003). Another intriguing issue is that while pathophysiological functions of SESN proteins are quite well documented, little is known about their molecular structure and the catalytic motif. The crystal structure was revealed only for human SESN2 protein and has shown two functionally distinct catalytic domains (Kim et al. 2015). These unique structural features of the SESN2 protein can integrate two seemingly unrelated, but physiologically important functions: the reduction of reactive oxygen species (ROS) and the inhibition of the mechanistic target of rapamycin complex 1 (mTORC1) (Kim et al. 2015). Kim et al. (2015) have reported that the SESN2 protein belongs to the family of globin-like α-helix-fold protein and contains three domains: N-terminal domain (Sesn-A), C-terminal domain (Sesn-C) and the flexible loop linker domain (Sesn-B). The Sesn-A domain reduces alkyl hydroperoxide radicals through its helix–turn–helix oxidoreductase motif. The motif in the helix–loop region of the Sesn-C domain is critical for the physical interaction with the GATOR2 complex and the subsequent modulation of mTORC1 signalling. The function of a flexible loop linker Sesn-B domain is not yet known. All three domains, Sesn-A, Sesn-B and Sesn-C, are separated by two unstructured flexible linker regions (Fig. 1) (Kim et al. 2015).

In the following section of the article, the structural insight into SESNs’ antioxidant function will be elaborated on. As a family of stress-inducible proteins, SESNs are widely expressed in the adult tissue. However, tissue-specific gene expression level of SESN proteins has been observed (Peeters et al. 2003). Among the three SESN proteins, SESN1 is predominantly expressed in the skeletal and cardiac muscle that shed new light on the physiological roles of SESNs in response to physical activity (Peeters et al. 2003; Kim et al. 2020b). The low expression of SESN1 was observed in the intestines, leucocytes, thymus and the spleen (Peeters et al. 2003). The SESN3 protein is expressed in the skeletal muscle, however, to a lesser extent than SESN1. Moreover, SESN3 is expressed in the intestine, liver, kidney, colon and the brain tissue (Peeters et al. 2003). The expression of the SESN2 protein is characteristic of colon and hepatocytes (Lee et al. 2012).

The diverse set of physiological roles that SESN proteins perform is crucial for metabolic homeostasis. Once induced, SESNs regulate multiple signalling pathways, such as AMP-dependent protein kinase (AMPK), mTORC1, nuclear factor erythroid 2-related factor 2 (Nrf2), mitogen-activated protein kinases (MAPKs) and transforming growth factor β (TGF-β) (Wempe et al. 2010; Bae et al. 2013; Lee et al. 2013; Kim et al. 2014). SESN proteins received special attention in the field of regulation of the ROS level in the cell and its interplay with the systems determining energy and nutrient homeostasis. ROS are a by-product of cellular metabolic processes that consume oxygen (He and Jiang 2016). Although the mitochondrial electron transport system (ETS) is very efficient, some electrons escape the ETS and react with molecular oxygen to form superoxide radicals. Superoxide radicals are also produced by the cytochrome 450 monooxygenase system in hepatocytes (Conklin 2004; Kozak et al. 2020). Thus, metabolic active cells constantly produce ROS that work as signalling molecules and interact with cellular macromolecules, including DNA, proteins and lipids (He and Jiang 2016; Kozak et al. 2020). Moreover, inefficient antioxidant systems primarily consisting of superoxide dismutase (SOD), catalase (CAT), glutathione peroxidases (GPxs), thioredoxin (Trx) and SESN proteins lead to abnormal accumulation of ROS. Consequently, ROS accumulation leads to the dysregulation of intracellular signalling pathways controlling cell proliferation and survival, cell motility and invasiveness as well as angiogenesis, apoptosis and anoikis that are implicated in tumour onset and progression (Fiaschi and Chiarugi 2012; Kozak et al. 2020). In cancerous cells, the elevated ROS level is a consequence of vigorous metabolism, increasing proliferation rate as well as the activation of oncogenes and impairment of the cell’s ability to detoxify ROS (Kozak et al. 2020). Paradoxically, excessive accumulation of ROS in cancerous cells can cause apoptosis. Therefore, the ROS generation approach is a potential anti-cancer therapeutic opportunity in which the SESN proteins could play a key role as molecules induced by oxidative stress (Kozak et al. 2020). The major theme of this review is to demonstrate the association between SESN proteins as biomarkers and therapeutic targets in cancer, an ROS-associated disorder. We hope this review will be helpful to researchers to behold SESN proteins as treatment modalities for cancer therapy and encourage scientists to delve deeper into the interactions between SESNs and ROS.

## Regulation of SESNs expression

SESN1, 2 and 3 belong to conserved stress-responsive proteins whose expression is mostly regulated by p53 and the forkhead transcription factor (FoxO) (Budanov et al. 2010). However, depending on the cell’s condition, other stress-inducible transcription factors regulate the expression of SESNs including Nrf2, the hypoxia inducible factor (HIF)-1α and JNK/c-Jun transcription factors (Bae et al. 2013; Zhang et al. 2013; Shi et al. 2016). SESN1 expression is under control of p53 tumour suppressor signalling pathway that is activated by excessive genotoxic damage (Lee et al. 2013). Interestingly, SESN1 was a member of Sestrin family firstly discovered as a p53-inducible gene (Velasco-Miguel et al. 1999). The regulation of SESN2 expression is only partially p53 dependent and other induction mechanisms are implicated. DNA damaging treatments, such as gamma radiation, UV radiation or doxorubicin, induce SESN2 expression under p53 regulation. However, prolonged hypoxia or oxidative stress induce SESN2 expression in a p53-independet manner (Budanov et al. 2002). One of the alternative mechanisms underlying SESN2 induction was discovered by Shin and colleagues (Shin et al. 2012). The authors revealed a specific role of Nrf2 in SESN2 expression induction. Additionally, they demonstrated that SESN2 was fundamentally required for Nrf2-mediated oxidative stress response pathway in the cell culture and in an animal model (Shin et al. 2012). Therefore, Nrf2 works as an upstream regulator and the downstream effector of SESN2. In another study, Zhang and colleagues revealed that C-Jun NH2-terminal kinase (JNK), which is commonly activated in cancer, is involved in the regulation of SESN2 transcription during autophagy (Zhang et al. 2013). More recently, it has been shown that SESN2 expression is regulated by activating the transcription factor 4 (ATF4) under endoplasmic reticulum (ER) stress condition triggered by nelfinavir and bortezomib in various cancer cell lines, HeLa, MDA-MB-45 and OVCAR3 (Brüning et al. 2013). In the same article, the authors suggest the association between antioxidant activity of SESN2 against ER stress and oxidative stress-inducing nelfinavir and bortezomib (Brüning et al. 2013). In contrast, Park et al. (2014) observed that SESN2 expression is induced by an ER stress-activated transcription factor CCAAT-enhancer-binding protein beta (c/EBPβ). However, the authors concentrate on chronic ER stress induced by excessive hepatic fat accumulation during obesity and non-alcoholic fatty liver disease instead of anti-cancer drugs activity (Park et al. 2014). The very first reports that SESN2 is upregulated by hypoxia, a severe metabolic stress condition, in a p53-independent manner, came from Budanov and colleagues’ experiments from 2002 (Budanov et al. 2002). Since that time, the implication of SESN in hypoxia has received much more interest. Some experiments have shown that SESN2 could be activated by HIF-1α under hypoxia conditions. Shi and colleagues have shown that HIF-1α induced the activation of SESN2 in severe hypoxic ischaemic (HI), but not in moderate HI in neonatal rats. Another study supporting the hypothesis that SESN2 is upregulated in an HIF-1α-dependent manner was performed by Essler et al. (2009). The authors evaluated the significance of hypoxia and nitric oxide (NO) on the transcription regulation of various genes involving hypoxia and oxidative stress in a macrophage cell line. Moreover, they have shown that SESN2 is not only regulated by HIF-1α, but also act as defence mechanism to combat hypoxia (Essler et al. 2009). The last member of SESN proteins family is SESN3, whose expression is regulated by the forkhead box O (FOXO) family of transcription factors in response to oxidative stress induced by the accumulation of ROS. The findings from the study of Nogueira and colleagues demonstrate that the expression of SESN3 is highly induced by activated FOXO3 and could contribute to the regulation of intracellular ROS and to resistance to oxidative stress (Nogueira et al. 2008). These findings are in agreement with the studies of Hagenbuchner and colleagues, who demonstrated that the induction of SESN3 by FOXO3 causes a transitory decline in the production of ROS and delays FOXO3-induced cell death of neuronal cells (Hagenbuchner et al. 2012). Therefore, SESN proteins may serve as key molecules for regulating oxidative stress induced cellular response by multiple signalling pathways.

## Antioxidant functions of SESNs

The overexpression of SESN proteins protects cells from death induced by hypoxia, glucose deprivation or hydroxyl peroxidase, suggesting an antioxidant function of SESNs. Initially, the biochemical mechanism underlying the antioxidant function of SESNs was connected with the activity of cysteine sulphinic acid reductase, which was necessary to maintain peroxiredoxins (Prxs) in an active form (Budanov et al. 2004). However, experimental attempts have not shown sulphinic acid’s reductase activity of SESN proteins (Woo et al. 2009). Nevertheless, the crystal structure of SESN2 revealed that Sesn-A domain exhibits the helix–turn–helix oxidoreductase motif that makes SESN2 protein a functional antioxidant enzyme (Kim et al. 2015). The functional experiments have revealed that SESN2 efficiently eliminates bulky hydrophobic ROS, but not small hydrophilic ROS (Kim et al. 2015). Despite the tremendous controversy over whether SESN2 indeed possesses oxidoreductase activity, SESN proteins protect cells from oxidative stress by two main pathways: inhibiting mTORC1 and promoting p62 autophagic degradation of Kelch-like ECH-associated protein 1 (Keap1), leading to the activation of Nrf2 transcription factor.

### Structural insights into SESNs’ antioxidant function

**Fig. 2:**
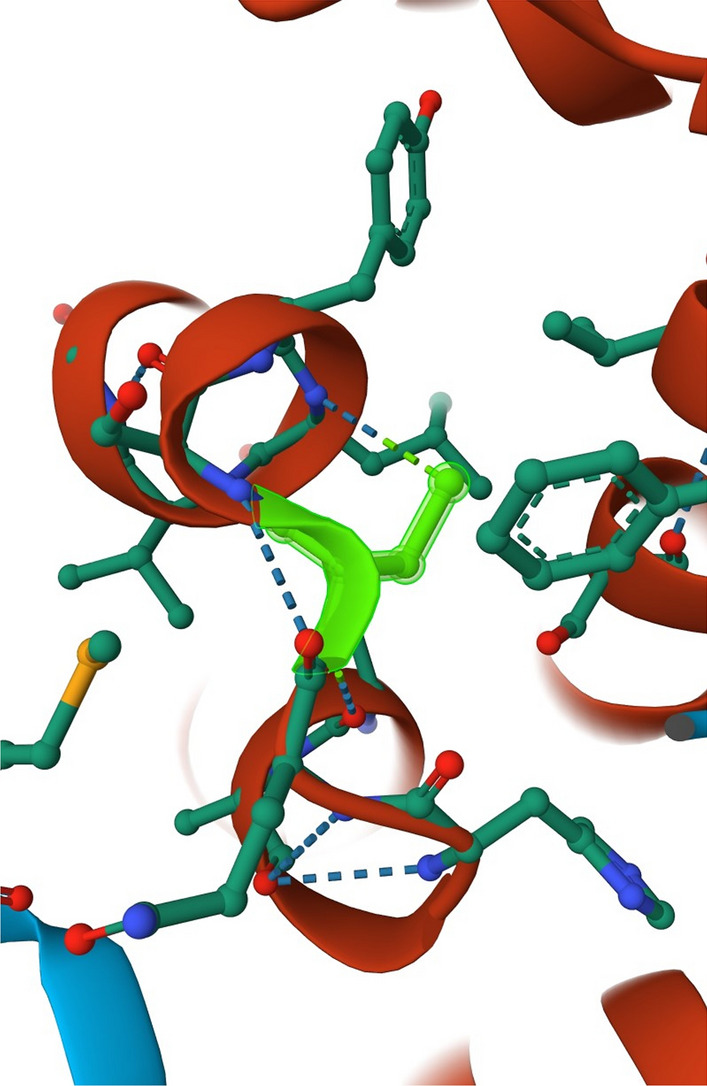
Catalytic cysteine 125. Sesn-A domain of SESN2 protein with catalytic cysteine (Cys125) is highlighted in green. Illustrations of the catalytic Cys125 used in the figure were generated with RCSB Protein Data Bank (RCSB PDB)

Crystallographic studies have revealed that SESN2 protein has two subdomains: A and C, which, structurally, are similar to an uncharacterized protein YP_296737.1 (PDB ID: 2PRR) in *Ralstonia eutropha* JMP134 and AhpD protein in *Mycobacterium tuberculosis* (Kim et al. 2015). Both of these proteins belong to the family of alkylohydroperoxidases. AhpD takes part in enzymatic regeneration of peroxiredoxin AhpC (bacterial counterpart of Prx), which is oxidized and, thus, inactivated during the reduction of peroxides and reactive nitrogen species (RNS). Structural similarities of AhpD and SESN2 suggest that SESN2 could also possess some enzymatic activity for Prx regeneration and/or directly towards ROS. Moreover, all three proteins exhibit the helix–turn–helix oxidoreductase motif with, well preserved within the Sesn-A domain, catalytic cysteine (Cys125) and residues in the proton delay system of AhpD-family oxidoreductases (Tyr127 and His132) (Fig. 2) (Kim et al. 2015).

Similar structural resemblance SESN2 protein to AhpD in a sense of catalytic Cys125 was proved by Budanov et al. (2004). As proposed by a different research group, enzymatic activity of SESN2 by homology to AhpD is based on the regulation of ROS homeostasis by regeneration of Pxr. It is thought that SESN2 is an active alkylohydroperoxide-detoxifying enzyme with only one Cys125 in the active catalytic site, and thus SESN2 enzymatic mechanism is different from AhpD that contains two cysteines in the catalytic site (Budanov et al. 2004; Kim et al. 2015). Further structural analysis of Sesn-A domain proved that Cys125 has hydrophobic surroundings, and thus could determine SESN2 enzymatic activity towards hydrophobic ROS such as alkylohydroperoxides (Kim et al. 2015).

**Fig. 3:**
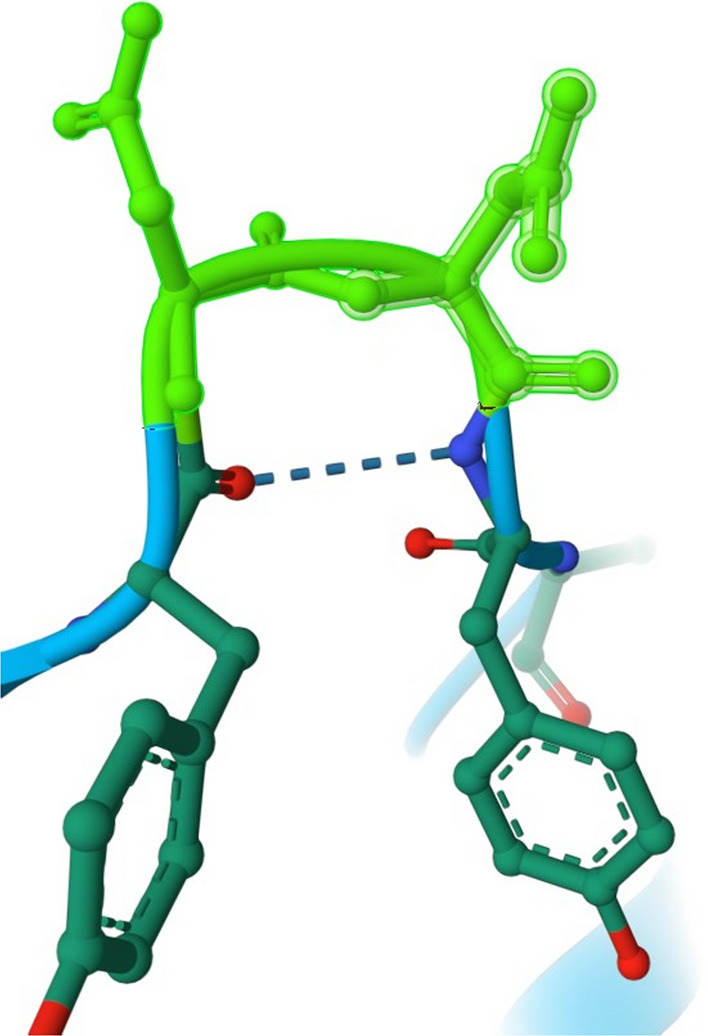
DD-motif of Sesn-C domain. Sesn-C domain of SESN2 protein with amino acid residues Asp406 and Asp407, which form the DD-motif. Asp406 and Asp407 are highlighted in green. Illustrations of the DD-motif used in the figure were generated with RCSB Protein Data Bank (RCSB PDB)

To understand the regulatory function of SESN 2 protein towards mTORC1, it is worth taking a closer look at the Sesn-C domain, which was revealed by crystallographic studies of Kim et al. (2015), so far the only study presenting the crystallographic structure of any SESN protein. As the authors have reported, the Sesn-C domain has a helix–loop structure instead of the helix–turn–helix motif. Presumably in the Sesn-C domain, amino acid residues Asp406 and Asp407 play a key role in the inhibition of mTORC1, which form the DD-motif responsible for the GATOR2 binding (Fig. 3) (Kim et al. 2015).

Based on these findings, we could speculate that SESN2 protein has two independently working domains, which could explain such diverse functionalities of SESN2 proteins. The open issue is that the crystal structure of SESN2 protein is also characteristic for the SESN1 and SESN3 proteins. It is observed that the Sesn-C domain sequence is conserved among all SESN proteins that suggest its importance for the mTORC1-regulating function (Kim et al. 2015).

Another study under the structural fundamentals of SESN2 protein towards mTORC1 regulation has revealed the importance of charged residues Glu451 and Arg390. These amino acids are present on the C-terminal end of the SESN2 protein where they form a single pocket critical for leucine binding. Moreover, to selectively bind the leucine, the base of the pocket is hydrophobic with residues Leu389, Trp444 and Phe447 (Saxton et al. 2016). Leucine is a well-documented amino acid promoting important physiological processes, including the enhancement of protein synthesis in the skeletal muscle tissue, adipose tissue and placental cells through mTORC1 activation; however, the detailed mechanism has been unknown. Moreover, leucine promotes glucose uptake, mitochondrial biogenesis and fatty acid oxidation, the processes that produce energy for protein synthesis, but it inhibits protein degradation (Duan et al. 2016). In close proximity to the leucine-binding pocket, key residues—Asp406 and Asp407 (DD-motif)—are located for GATOR2–SESN2 interactions. The authors have suggested that the close proximity of leucine-binding pocket and GATOR2-binding site is of crucial importance for GATOR2 realising form SESN2 inhibition when leucine is bound SESN2 (Saxton et al. 2016). The physiological consequence of GATOR2 dissociation from SESN2 will be discussed in the next section. Nevertheless, this study has provided a molecular explanation of how the SESN2 specifically detects leucine that finally disrupts SESN2–GATOR2 interaction and causes mTORC1 activation, thus enhancing protein synthesis and cell growth. These structural findings underline the discovery of the selective brain mTORC1 activator NV-5138, which binds SESN2 protein the way leucine does (Sengupta et al. 2019). The identification of NV-5138 is of considerable importance for the validation of SESN2 and SESN1 as new therapeutic targets for the modulation of the activity of mTORC1 via an upstream regulatory mechanism. In contrast to the plethora of experiments that almost exclusively centred on developing mTOR inhibitors working in anti-cancer therapy, Sengupta et al. (2019) focus on the mTOR activator needed in the therapy of the central nervous system (CNS) diseases including depression and conditions linked to cognition, learning and memory.

### AMPK and mTORC1 signalling

**Fig. 4:**
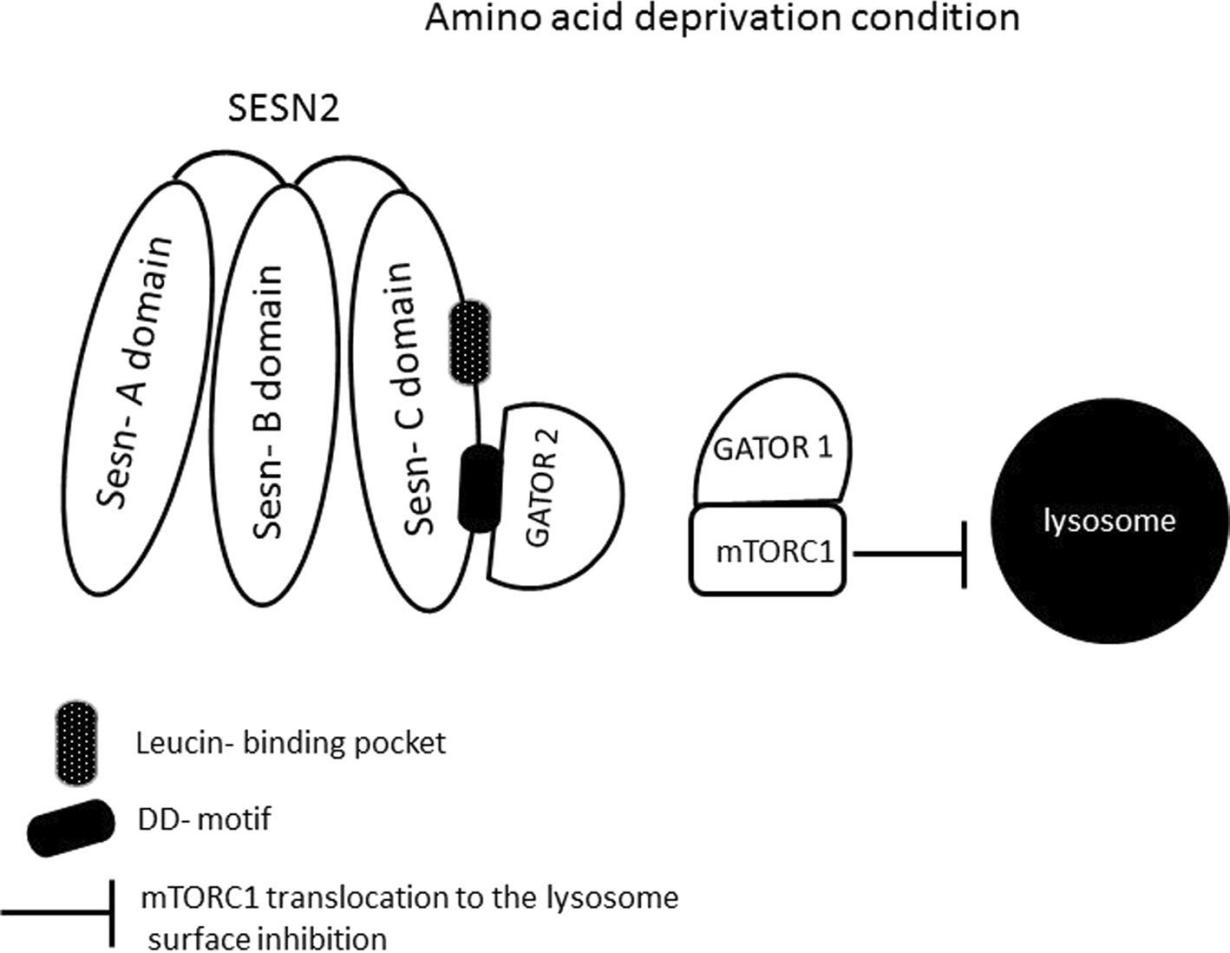
A model explaining SESN2 protein interactions under amino acids deprivation condition

SESN proteins are mostly upregulated in cells under oxidative stress, hypoxia and DNA damage conditions. SESN proteins have the potency to suppress the activity of mTORC1 through the activation of AMPK under genotoxic and metabolic stress (Budanov and Karin 2008). The mTORC1 complex is responsible for the control of cell growth and protein synthesis. The activity of mTORC1 is positively regulated by the growth factors and inhibited by nutrient limitation, hypoxia and DNA damage (Budanov and Karin 2008; Mihaylova and Shaw 2011). The positive and negative control of mTORC1 activity is exerted through the tuberous sclerosis complex (TSC), TSC1:TSC2 complex, whose TSC2 subunit serves as a GTPase activating protein (GAP) for the small GTPase Rheb, which activates mTORC1. GAPs stimulate GTP hydrolysis or stimulates guanine nucleotide exchange factors (GEFs) that facilitate GDP dissociation or stimulates guanine nucleotide dissociation inhibitors (GDIs) that prevent GDP dissociation. The TSC2 activity towards GTPase Rheb relies on GTP hydrolysis converting Rheb from GTP-bound active state to GDP-bound inactive state (Peng et al. 2014). GDP-bound Rheb cannot activate mTORC1. Importantly, TSC2 activity is regulated by AMPK (Budanov and Karin 2008). AMPK is a heterotrimeric complex comprising two α (α1, α2), two β (β1, β2) and three γ (γ1, γ2, and γ3) subunits that regulate metabolic activities influencing cell growth and proliferation. In response to stress conditions, AMPK phosphorylates numerous substrates to restore energy homeostasis and thus help to protect the cells against stress-induced apoptosis (Mihaylova and Shaw 2011). The most thoroughly described mechanism by which AMPK regulates cell growth is via suppression of the mTORC1 where SESN1 and SESN2 proteins play an important role. Budanov and Karin revealed that SESN1 and SESN2 activate AMPK through direct interaction and stimulate its kinase activity towards TSC2, which is present in a complex with AMPK and SESN1 and SESN2. Thus, TSC2 phosphorylation stimulates its GAP activity causing hydrolysis of GTP bound to Rheb. Rheb–GDP inactive complex cannot activate mTORC1, thereby phosphorylation of TSC2 indirectly inhibits mTORC1 (Budanov and Karin 2008). A multiprotein complex mTORC1 functions as an integrating centre of environmental and metabolic signals with protein synthesis, lipids anabolism and, in consequence, control of cellular growth and suppression of autophagy (Cordani et al. 2019). When activated, mTORC1 phosphorylates substrates that potentiate anabolic processes and limit catabolic ones, such as autophagy. Thus, mTORC1 senses nutrient and energy levels and protects proliferating cells from energetic stress-induced death in the case of nutrient depletion conditions (Byun et al. 2017). However, a detailed description of mTORC1 is beyond the scope of this article and has not been reviewed. To get acquainted with the detailed characteristics of mTOR, we refer to the excellent scientific papers available in the databases of widely read journals. It is speculated that SESN proteins can work as cellular sensors of amino acids in the cell microenvironment and, in this sense, appear to be negative regulators of mTORC1 activity (Chantranupong et al. 2014; Saxton et al. 2016). However, the detailed mechanism is complex and depends on the biological context. Thus, the role of SESNs as amino acids’ sensors needs further clarification. An interesting piece of information about the SESNs regulation of mTORC1 in amino acid-sensitive fashion comes from the work of Chantranupong et al. (2014). The authors speculated that SESN2 inhibits mTORC1 activity through the modulation of GATOR1–GATOR2 in amino acid deprivation conditions (Chantranupong et al. 2014). SESN2 binds to the GATOR2 complex directly by the DD-motif present on the Sesn-C domain and liberates GATOR1 from GATOR2-mediated inhibition, leading to mTORC1 inhibition (Chantranupong et al. 2014; Kim et al. 2015). SESN2 requires GATOR1 to inhibit mTORC1 translocation to the lysosome surface where it is activated by the previously described Rheb GTPase (Peng et al. 2014; Chantranupong et al. 2014). At this point, it is worth explaining that amino acids appear to signal mTORC1 through the heterodimeric Rag GTPases, which consist of Rag A or Rag B bound to Rag C or Rag D. The active Rag A/B-GTP and Rag C/D-GDP complex promotes mTORC1 translocation to the lysosome surface where Rheb also resides and activates mTORC1 (Chantranupong et al. 2014). The GATOR1 complex functions as a GAP for Rag A/B GTPases and, thereby, inhibits the localization of mTORC1 at lysosomes (Peng et al. 2014; Chantranupong et al. 2014; Kim et al. 2015). Moreover, GATOR1 interacts with GATOR2 complex and together they regulate mTORC1 in response to amino acids deprivation in AMPK-independent manner, but SESN2-dependent fashion (Fig. 4).

Altogether, the presented results suggest a regulatory role for SESN2 in signalling amino acid sufficiency to mTORC1 in the GATOR1 and GATOR2 interacting loop. We paid special attention to figuring out the SESN–mTORC1 interactions because it is known that the mTORC1 deregulation occurs in cancer and promotes cancer cell survival and progression. In several human cancer cell lines, chronic activation of the mTORC1 complex leads to increased SESN2 expression (Lee et al. 2013). Thus, understanding the modulatory role of SESN proteins in the mTORC1 signalling pathway may contribute to predicting the therapeutic response to the anti-cancer drug treatment or even may work as a molecular target for new anti-cancer therapies. It is known that the best-known function of SESN2 is the inhibition of the mTORC1. Surprisingly, the recent work of Byun et al. (2017) suggests a positive feedback loop between SESN2 and mTORC2 which is necessary to suppress the activity of mTORC1 in glutamine-depleted non-small cell lung cancer (NSCLC). This work demonstrates that SESN2 regulates mTORC1 and mTORC2 in different ways, finally leading to redox balance and lung cancer cells survival under glutamine-depleted conditions (Byun et al. 2017). The considerable importance of this work lies in the combination of cancer cells’ nutrient stress and the prevention of excessive accumulation of ROS with SESN2, mTORC1 and mTORC2. The metabolic reprograming that implicates SESN2, mTORC1 and mTORC2, enables lung cancer cells survival under glutamate deprivation conditions. Thus, understanding, the interactions between SESN2 and mTORC2 components at the molecular level, and their impact on mTORC1, may help to overcome this cancerous adaptive mechanism. It seems that independently of redox activity, SESN proteins lead to AMPK-dependent inhibition of mTOR signalling and link genotoxic stress to mTOR regulation. The metabolic adaptation of cancer cells to energy starvation is critical for their survival and understanding of this mechanism along with the role of SESN proteins will be important to develop effective anti-cancer therapies. The potential tumour-suppressing effects of SESN2 in colorectal cancer cell lines, tissue and a xenograft mouse model were speculated by Wei and co- workers. The authors elucidated the molecular mechanism behind the potential contribution of SESN2 to counteract colorectal cancer (CRC) via the activation of AMPK, thereby down-regulating the mTORC1 pathway (Wei et al. 2017). Their findings indicated that SESN2 overexpression suppresses proliferation and activates apoptosis in CRC cell lines and also inhibits the growth of CRC xenografts in nude mice (Wei et al. 2017). Another study demonstrating the correlation between SESN protein and AMPK has been performed by Cordani et al. The authors showed that mutant p53 proteins can stimulate their oncogenic pro-oxidant conditions through the inhibition of SESN1 expression and, consequently, can inhibit the SESN1–AMPK complex and downstream protein targets of AMPK (Cordani et al. 2018). Beyond the regulation of cellular metabolism, AMPK is considered to control the expression of several mitochondrial enzymes and proteins, including proliferator-activated receptor gamma coactivator-1 alpha (PGC-1α) resulting in an increase of intracellular ROS levels, which sustain an invasive phenotype of cancer cells (Handschin et al. 2003; Jager et al. 2007; Luo et al. 2016; Rabinovitch et al. 2017). Importantly, the inhibition of AMPK signalling is involved in the pro-oxidant role of mutant p53 in cancer cells stimulating mitochondrial ROS production that could produce genomic DNA damage and genomic instability, which are typical hallmarks of cancer cells bearing mutant p53 protein (Cordani et al. 2018). UV radiation is considered to induce DNA damage and cellular stress, which activates p53 protein. P53 is an important cellular gatekeeper that maintains genomic stability by inducing DNA repair or apoptosis (Mlitz et al. 2016). Moreover, p53 has the potency to regulate the expression of SESN1 protein (Velasco-Miguel et al. 1999). Mlitz et al. (2016) explained that SESN1 and SESN2 are upregulated during the UVB stress response of skin cells and suggested that both SESN1 and SESN2 proteins’ upregulation depends on p53. Furthermore, they suggested that UVB stress of skin cells alters mTORC1-dependent processes to restore cellular homeostasis. Thus, signals from both, SESN1 and SESN2, are likely to contribute to the regulation of mTORC1 (Mlitz et al. 2016).

### Autophagy

Autophagy is characterized as a complex intracellular process comprising the degradation and recycling of macromolecules and worn-out malfunctioning organelles (Ornatowski et al. 2020). Thus, autophagy is essential for maintaining cellular homeostasis and is a highly regulated mechanism. A major stimulus for autophagy activation in cells is the inhibition of mTORC1 signalling and/or activation of AMPK through phosphorylation of an autophagy-related protein (ATG) and an unc-51-like autophagy-activating kinase 1 (ULK1) protein (Kim and Lee 2014). The final step of autophagy is the formation of an autophagosome and its fusion with the lysosome causing the degradation of the autophagosome cargo (Kma and Baruah 2021). This clearance system in cells is anticipated to maintain the delicate balance between excessive buildup of the loss-function biomolecules and organelles and eliminating the ones that are misfolded or damaged. Given that oxidative stress leads to the damage of the cellular components which, in consequence, lost their function, autophagy is also induced by abnormal ROS accumulation and is found to contribute to antioxidant function (Ornatowski et al. 2020; Kma and Baruah 2021; Li et al. 2021). This is achieved by the release and activation of Nrf2, a transcription factor that orchestrates antioxidant response. Ubiquitin-binding protein p62 enhances Keap1–Nrf2 dissociation and facilitates the degradation of Keap1 by p62-dependent autophagy, thus promoting Keap-1 degradation and Nrf2 activation. These interactions between p62, Keap-1 and Nrf2 lead to further stimulating the antioxidant response (Bae et al. 2013; Ornatowski et al. 2020; Kma and Baruah 2021). In addition, SESN proteins also promote p62-dependent autophagic degradation of Keap-1 by direct interaction with p62 and their activation (Bae et al. 2013; Ornatowski et al. 2020). Hence, it seems to be interesting to explore whether the autophagy signalling pathway interacts with SESN proteins. This will be particularly important for the study of the role of autophagy, ROS and SESN proteins in cancer cells that are characterized by persistently elevated ROS levels because of increased metabolic rate, malfunctioned mitochondria and disturbances in a plethora of cell signalling pathways. Among these signalling pathway, there is mTORC1 and AMPK that integrate the cellular response to oxidative stress with control of energy management and cell growth. This concept might be controversial, and it is likely reliant on the type and stage of the cancer, together with extending oxidative stress and the cost of growth and survival of cancer cells in such conditions. Several reports indicate that SESNs are the regulators of autophagy. For example, Bea and colleagues have found that forced expression, mostly of SESN2 but also SESN1, promotes the autophagic degradation of Keap1 and, thereby, stabilizes and upregulates Nrf2 signalling, leading to the induction of genes for antioxidant enzymes in mice hepatocytes (Bae et al. 2013). It has been suggested that SESNs proteins serve as scaffold proteins that strengthen the weak association of Keap1 with p62. Thus, SESN2 and SESN1 have been identified as positive regulators of the Nrf2 pathway which protects the mouse liver from oxidative injury (Bae et al. 2013). At the same time, the p62 and SESN2 genes are targets of Nrf2. Thus, the accumulation of p62 and SESN2 and the activation of Nrf2 constitute positive feedback loops (Shin et al. 2012; Rhee and Bae 2015). Another study indicates that SESN2 is a positive regulator of autophagy in the cellular model of Parkinson’s disease (Hou et al. 2015). It was shown that SESN2 enhanced autophagy in an AMPK-dependent fashion, as the overexpression of SESN2 activated AMPK. Moreover, in the same experiments, it was found that autophagy was induced with a concomitant increase p62 (Hou et al. 2015). Autophagy can be triggered by multiple types of cellular stress and nutrient depletion is one of them (Maiuri et al. 2009; Kim and Lee 2014). The molecular pathway that links nutrient depletion to autophagy involves the activation of AMPK, which in turn inhibits mTORC1, the autophagy repressive kinase (Maiuri et al. 2009). Moreover, SESN proteins inhibit mTORC1 through the activation of AMPK (Budanov and Karin 2008). These findings support the hypothesis that SESN proteins might play an important role as a bridge between autophagy and cellular redox metabolism. Recent investigations have shown that SESN2 is also a substrate of ULK1 and its phosphorylation mediates mTORC1 inhibition, highlighting the complex cross talk between the SESN proteins family and autophagy (Kimball et al. 2016; Kim et al. 2020a). One of the best-recognized ways of cancer cells elimination upon chemotherapy is apoptosis often caused by autophagy. However, there is considerable controversy about the role of autophagy regarding the regulation of chemoresistance. Potentiation of autophagy could be used by cancerous cells as a defence mechanism against chemotherapy agents, thus leading to chemoresistance. Tang et al. (2021) have found that osteosarcoma cell lines HOS, MG-63 and 143B treated with cisplatin, doxorubicin and methotrexate show increased expression of SESN2 that enhances autophagy and reduces the sensitivity of osteosarcoma cells to chemotherapeutic agents by inhibiting apoptosis. Such phenomenon of SESN2 activity emerges from increased endoplasmic stress induced by chemotherapeutic agents in osteosarcoma cell lines. The authors have speculated that increased level of SESN2 inhibits the endoplasmic stress signalling pathway PERK–eIF2α–CHOP following the treatment with cisplatin, doxorubicin and methotrexate, thereby upregulating autophagy in osteosarcoma cells (Tang et al. 2021). The experimental silencing of SESN2 increased the sensitivity of osteosarcoma cells to chemotherapeutic agents and increased their apoptosis rate. In conclusion, these experiments suggest that SESN2 may serve as a pro-cancerous protein increasing cancer cells chemoresistance in specific circumstances such as endoplasmic stress. At the same time, elevated SESN2 level could act as a predicting factor in osteosarcoma cells’ response to cisplatin, doxorubicin and methotrexate. Therefore, it seems reasonable to put an effort into further research to find the key chemotherapy response predictor that could change the direction of the cellular response from resistant into sensitive to anti-cancer therapy. Based on the presented results, SESN2 protein could have such potential to be a modulator of osteosarcoma cell response to chemotherapy. In contrast to Tang’s results, Yen et al. (2018) revealed that tanshinone IIA (TIIA) isolated from the herb *Salvia miltiorrhiza* induces autophagy in an SESN2-dependent manner that inhibits osteosarcoma cell lines MG-63 and 143B growth. However, the observed anti-cancerous effect of TIIA is mediated by HGK/JNK1/C-Jun signalling cascades, leading to SESN2 expression and autophagy induction. Such conflicting cellular responses to autophagy induction in an SESN2-dependent fashion could be explained by an activation of distinct signalling pathways reported in the aforementioned studies.

In summary, various aspects of the interplay between the autophagy cellular pathway and modulatory activities of SESN proteins have been shown. The discrepancy between the role of autophagy in cancer may be similar to the role of oxidative stress in tumorigenesis. The moderate autophagy may acts as self-protection mechanism against cytotoxicity of anti-cancer drugs, while consequent excessive autophagy may lead to cancer cell death (Cirstea et al. 2010; Tomic et al. 2011). Another important source of autophagy ambiguity could be cancer-specific response to chemotherapeutics. The response may be influenced by the different autophagic pathways utilized and by the type of cancer cells and possibly stage of tumour. It seems that the possibility to modulate autophagy by SESN proteins may represent a promising therapeutic approach to treating different types of cancer. However, further large-scale studies are required to prove the potential clinical utility of SESN proteins as a modulator of autophagy. On the other hand, SESN proteins have several different functions, and regulation of autophagy is only one of them.

## Expression profile of SESNs in various cancers

Accumulating evidence has reported that most of cancer tissues and cancerous cell lines are accompanied by a remarkable change of SESN proteins’ expression level. The aberrant expression of SESN proteins is associated with different cellular processes such as proliferation, invasion, metastasis, autophagy, oxidative stress apoptosis and drug resistance. A decreased expression of SESN2 has been showed in human colorectal cancer (CRC) tissues and HT-29, SW480, SW620 and LoVo cell lines. Moreover, the expression of SESN2 in SW620 and LoVo cells, derived from the metastatic site of CRC, was significantly lower than that in the HT‑29 and SW480 cells, derived from the primary lesion of CRC (Wei et al. 2015). These data could suggest some association between tumour stage and activity of SESN protein, which has been confirmed by experiments on human CRC tissues. The lower expression of SESN2 was associated with advanced tumour stage, lymph node and liver metastasis and vascular invasion (Wei et al. 2015). Thus, low expression of SESN2 in CRC may increase oxidative stress and aggravate tumour metastasis. Evidence suggests that increasing ROS level in cancer cells is involved in the EMT process by cytoskeleton remodelling, regulation of ECM (extracellular matrix) remodelling, cell–cell junctions’ regulation and regulation of cell motility (Kozak et al. 2020). Another important issue is that excessive accumulation of ROS can cause cancer cell apoptosis, resulting in antitumour effects. Therefore, the ROS generation approach can be applied to the treatment of cancer cells. In this respect, it paradoxically seems that cancer cells may die by the same system they require for progression (Kozak et al. 2020). More importantly, colorectal cancers are poorly vascularized, and therefore easily exposed to hypoxic conditions that are primarily controlled by the transcription factor HIF-1α. Seo et al. (2016) took a closer look at the effect of SESN2 on signalling pathways altered by hypoxia in colon cancer cells. Their experiments revealed that experimentally overexpressed SESN2 inhibits HIF-1α accumulation in colon cancer cell lines HCT116 and HT29 exposed to hypoxic condition and prevents cancer cell metastasis. Moreover, the authors speculate that SESN2-mediated suppression of HIF-1α accumulation and cancer cell migration could have resulted from AMPK activation (Seo et al. 2016). The decreased expression of SESN2 in CRC tissue was reported by Ro and colleagues. Furthermore, they proved that SESN2 is a clinically relevant target of p53 during colon carcinogenesis, and downregulation of SESN2 induces mTORC1 hyperactivation, which subsequently allowed for prominent tumour overgrowth (Ro et al. 2016). In functional studies, SESN2 was overexpressed in p53-deficient HCT116 cells and SW480 cells, which have low SESN2 expression and high mTORC1 signalling. They proved that SESN2 attenuates cancer cell growth primarily through inhibition of a hyperactive mTORC1. Moreover, they treated SESN2-silenced RKO cells with two representative chemotherapeutic agents, 5-fluorouracil (5-FU) and irinotecan (CPT-11), to asses if SESN2 may be important for the responsiveness of colon cancer cells to chemotherapeutic treatments. Their results indicate that SESN2 loss and consequent mTORC1 upregulation may influence on RKO cells chemoresistance to 5-FU and CPT-11 (Ro et al. 2016). In line with these findings, Wei and co-workers revealed that the overexpression of SESN2 suppresses proliferation and activates apoptosis in CRC SW620, LoVo cell lines, and also inhibits the growth of CRC xenografts in nude mice. Their results suggest that the reduction in SESN2 levels in human CRC samples may contribute to CRC pathogenesis via the regulation of the AMPK/mTORC1 pathway (Wei et al. 2017). Low expression of SESN2 was also observed in primary invasive bladder cancer tissue and was associated with bladder cancer formation. Further, the restoration of a high level of SESN2 was sufficient for promoting autophagy and further mediating the inhibition of an anchorage-independent growth of human bladder cancer cells (Liang et al. 2016; Hua et al. 2018). Similarly, low expression of SESN2 protein was reported in LNCaP clone FGC, DU145 and PC3 cell lines compared to normal prostate epithelial cell line RWPE-1. PC3 cell line has been widely used in progressive stages of prostate cancer cell models, and it is more similar to some castration-resistant prostate cancers in clinical conditions. Additionally, the authors have shown that exogenous overexpression of SESN2 inhibits PC3 cell proliferation, sensitizes to ionizing radiation treatment and significantly increased PC3 cell apoptosis after ionizing radiation treatment (Fu et al. 2018). However, the molecular mechanism responsible for the observed SESN2 activity has not been elucidated. Likewise, in vitro overexpression of SESN2 was associated with the inhibition of the invasion, migration and proliferation of PANC-1 and CFPAC-1 pancreatic cell lines. The suggested mechanism behind SESN2 anti-cancerous activity is enhancing Nrf2/Keap1/HO-1/NQO-1 signalling pathway (Fu et al. 2021).

Interestingly, it was observed that SESN2 is upregulated in endometrial cancer (EC) tissue and correlated with shorter overall survival and disease-free survival in patients with endometrial cancer (Shin et al. 2020). The authors have speculated that elevated SESN2 protein level is induced upon chronic activation of mTORC1 in endometrial cancer cells. The transcriptional activation of SESN2 is one of the negative feedback mechanisms for inhibiting chronic activation of mTORC1 and preventing the positive effects of mTORC1 on cancer cells proliferation and progression (Shin et al. 2020). Complementary to these results, our recent study has shown that the expression of SESN2 was elevated in AN3CA, Ishikawa, RL-92-5 and KLE EC cell lines. However, among all the tested EC cell lines, the highest level of SESN2 was present in AN3CA cell line and the lowest one in KLE cell line. Since each of the tested EC cell lines represents a different stage of EC, SESN2 expression may be correlated with tumour stage and tumour metastasis (Kozak et al. 2019). Moreover, SESN3 expression was faint in the KLE and RL-95-2, while it was not detected in Ishikawa and AN3CA cell lines. Further, SESN1 was not expressed in any of the analysed endometrial cancer cell lines (Kozak et al. 2019). Interestingly, it was shown that SESN2 and SESN3, in a microRNA-200 family-dependent manner, influence anoikis resistance in EC cell lines (Kozak et al. 2019). In line with these findings, Zhu and co-workers mentioned that metastatic melanoma shows an increased expression of SESN2, which may serve as a predictor of poor prognosis. Similar results to cancer tissue sample were obtained in metastatic melanoma cell lines A375, A2058, UACC62, UACC257, 451Lu, and HTB67 (Zhu et al. 2020). The authors hypothesize that upregulated SESN2 may facilitate ECM-detached melanoma cells in anoikis resistance. Such phenomenon, as the authors suggested, could be attributed to detoxification of intracellular ROS level by SESN2 (Zhu et al. 2020).

There have been conflicting reports on the expression level of SESN proteins in lung cancer. Chen and colleagues have tested 210 primary non-small cell lung cancer (NSCLC) tissues among them 114 samples were characterized by low expression of SESN2 and 96 samples were characterized by high expression of SESN2. Moreover, low SESN2 expression was correlated with the characteristic of aggressive NSCLC including poor tumour differentiation, advanced TNM stage and lymph node metastasis, in contrast to high SESN2 expression. Further, patients with low SESN2 expression had worse overall survival compared with those with high SESN2 expression (Chen et al.). Thus, the expression level of SESN2 could be important in the observation of prognosis in NSCLC. Complementary to these results, Ding and co-workers have shown that the expression levels of the *SESN1* and/or *SESN2* genes are often diminished in human lung cancers and suppression of SESN1/2 may support tumour growth and/or progression through such mechanisms as stimulation of cell growth, proliferation, mutagenesis and angiogenesis. More importantly, the authors have speculated that SESN1 and SESN2 might play opposite roles in the regulation of early and late stages of lung carcinogenesis. They hypothesized that certain levels of SESN2 expression during the early steps of carcinogenesis might be required to support the activity of AKT kinase, the critical positive regulator of glucose transport, anabolism and cell viability that contribute to growth and proliferation of tumour cells. However, the inactivation of SESN1 and/or SESN2 in A549 cells accelerates cell proliferation and imparts resistance to cell death in response to glucose starvation (Ding et al. 2019).

Altogether, SESN proteins and SESN2 in particular are implicated in tumour onset and progression of the several types of human cancer. Their expression level could be used as a prognostic marker and clinicopathological characteristics of patients. However, the complex nature of cancer makes it difficult to judge whether SESN proteins are solely anti-cancerous or pro-cancerous and poses challenging questions about the role of SESNs in tumour biology. The potency of SESN proteins to cross talk with a plethora of regulating targets in a specific cellular context pose only a few obstacles to overcome before SESNs become an effective anti-cancer therapy. Nevertheless, we made the attempt to demonstrate the evidence for an eminent role of SESN proteins in anti-cancer therapy in the following section.

## SESNs in anti-cancer therapy

SESN are stress-inducible proteins that modulate the antioxidant and autophagy signalling pathways that protect cells from various harmful stimuli. Since perturbations in these pathways are implicated in cancer onset and development, SESNs might constitute potential novel therapeutic targets of broad interest. In this section, we discuss the contribution of SESNs regulatory networks to the pharmacological effects of different anti-cancer substances.

### Natural compounds regulating SESNs

Naturally occurring compounds from dietary sources are frequently investigated for their anti-cancer activities. Some of them are reported to exert their activities through direct modulation of SESN proteins expression. One of them is cucurbitacin B, a tetracyclic triterpenoid present in plants of the Cucurbitaceae family, i.e. pumpkins, gourds and squashes (Khan et al. 2016). It was shown that cucurbitacin B had antiproliferative effects on hepatocellular carcinoma cells, pancreatic cancer cells, breast cancer cells, leukaemia and lymphoma cell lines (Haritunians et al. 2008; Zhang et al. 2009; Iwanski et al. 2010; Aribi et al. 2013; Gupta and Srivastava 2014). Khan and co-workers have revealed that cucurbitacin B exhibits antiproliferative effects on human non-small cell lung cancer (NSCLC) cells. Via a functional analysis, they demonstrated that cucurbitacin B inhibits PI3K/mTOR and STAT-3 signalling pathway in conjunction with the activation of AMPKα in EGFR wild-type and mutant lung cancer cells. The authors suggest that such activity of cucurbitacin B could be achieved by increased SESN3 expression in lung cancer cells (Khan et al. 2016). According to the well-documented activity of SESN3 towards the inhibition of mTORC1 via the activation of AMPK, the proposed mechanism regarding cucurbitacin B is highly probable. Moreover, they found that that SESN3 has a prominent role in the induction of apoptosis in epidermal growth factor receptor (EGFR)-mutant cancer cells (Khan et al. 2016).

The implication of naturally occurring compounds in the regulation of SESN proteins was also demonstrated in bladder cancer. The two studies presented below have shown the induction of autophagy-dependent anti-cancer effect via upregulating SESN2 protein in human bladder cancer cells. More precisely, Liang and colleagues (Liang et al. 2016) took a closer look at the antitumour effect of isorhapontigenin (ISO), a new derivative of stilbene isolated from the Chinese herb *Gnetum cleistostachyum*. Their experiments revealed that ISO treatment induces autophagy effectively in human bladder cancer cells, which contributes to the inhibition of anchorage-independent growth of cancer cells. Interestingly, their findings indicated that ISO-mediated autophagy induction occurred in an SESN2-dependent manner (Liang et al. 2016). The speculated mechanism of increased SESN2 expression under ISO treatment is related to the MAPK8–JUN (mitogen-activated protein kinase 8) pathway. MAPK8 belongs to the stress-activated protein kinase MAPK family and, as the authors suggested, ISO treatment increased MAPK8–JUN phosphorylation in bladder cancer cells (Liang et al. 2016). MAPK8 is initially activated in response to a variety of stress signals and is implicated in many cellular events including apoptosis and autophagy (Shimizu et al. 2010). JUN is a well-known oncogene involved in cancer progression. JUN works as a transcription factor which plays a crucial role in signal transduction pathways and is involved in cell division, motility, adhesion and survival in both normal and cancer cells (Parsons and Parsons 2004; Kani et al. 2017). The authors have suggested that ISO treatment results in activation of JUN, which in turn upregulates SESN2 expression (Liang et al. 2016).

More recently, the inhibition of anchorage-independent growth and the induction of autophagy in an SESN2-dependent fashion were demonstrated in human high-grade invasive bladder cancer (BC) cells. The phenomenon, reported by Hua et al. (2018), occurs in BC cells under ChlA-F treatment. ChlA-F is a novel conformation derivative of cheliensisin A, styryl-lactone isolates that show potent anti-tumour potential in vivo and in vitro. The suggested mechanism behind SESN2 upregulation that the authors proposed is Sp1 transcription factor activation under ChlA-F treatment. Therefore, in contrast to Liang’s results, this study defines distinct mechanistic cascades that mediate the autophagic induction by upregulated SESN2. The aforementioned findings confirmed the importance of SESN2 as a positive regulator of autophagy.

SESN proteins hold great potential to become a beneficial target for completely novel compounds and also for well-known anti-cancer therapies. The potential antitumour activity of SESN proteins lies in their possibility to control ROS and regulate cell metabolism and autophagy. Complementary to these observations, Kim et al. (2013) reported that quercetin, which is a polyphenolic compound extracted from red onion and green tea, generates intracellular ROS and induces apoptosis through the inhibition of mTOR via SESN2 in HCT116 and HT-29 colon cancer cell lines. Moreover, they hypothesized that quercetin’s activity is accompanied by AMPK phosphorylation (Kim et al. 2013). Further experiments with quercetin and colon cancer cells revealed that induction of apoptosis by quercetin occurred through the activation of the AMPK/p38 signalling pathway and was dependent on SESN2, but independent of p53 (Kim et al. 2014).

Another study looked at phytochemicals present in rosemary, especially carnosol which has the potential to promote gastrointestinal health by activating the antioxidant SESN2 (Yan et al. 2018). Yan et al. (2018) hypothesized that carnosol is capable of inducing the Nrf2 network in colon cancer cells and upregulating SESN2 expression. Interestingly, low expression of SESN2 has been associated with advanced tumour stage, lymphatic invasion, metastasis, vascular invasion, liver metastasis and decreased survival rate. SESN2 deficiency in human colorectal cancer cells was also associated with decreased susceptibility to chemotherapeutic drugs (Wei et al. 2017). All of this suggests that the restoration of SESN2 expression could be beneficial for improving the therapeutic response to anti-cancer drugs.

The modulation of SESN2 expression by eupatilin, a flavone derived from *Artemisia asiatica*, has been shown by Jegal et al. (2016). They observed that eupatilin protects hepatocyte cells against arachidonic acid (AA) and iron-induced oxidative stress through SESN2-mediated autophagy induction. Moreover, the authors suggested that Nrf2 activation by eupatilin might contribute to SESN2 induction and that SESN2 induction by eupatilin might activate Nrf2. This finding is in agreement with the aforementioned positive feedback loops between SESN2 and Nrf2 (Shin et al. 2012; Rhee and Bae 2015).

Oxidative stress and ROS accumulation are implicated in tumour onset and progression (Kozak et al. 2020). Interestingly, increasing the ROS generation can make cancerous cells sensitive to chemotherapeutic interventions or other therapeutics that act by further augmenting the production of ROS (Conklin 2004; Fiaschi and Chiarugi 2012; Harris et al. 2015; Kozak et al. 2020). In line with these findings, Lin et al. (2018) performed a series of experiments on arsenic trioxide (ATO), a traditional Chinese medicine that can induce oxidative stress for the treatment of cancer cells. Puzzlingly, their findings have shown that ATO can also promote antioxidant activities by the upregulation of SESN2 in patient-derived primary S1 glioblastoma (GBM) cells, the U87MG glioma cell line, the human lung adenocarcinoma H1299 cell line and the A549 cell line. Moreover, they suggested that ATO-mediated suppression of miR-182 is inversely correlated with the expression of anti-oxidative SESN2 protein level that protects the cells from oxidative stress (Lin et al. 2018). A dysregulation of intracellular ROS homeostasis contributes to inhibition or induction of miRNAs expression profile which generates subsequent biological effects through the regulation of their target genes. Therefore, it is of great importance to take a closer look at the cross talk between microRNA, oxidative stress and the antioxidant system when designing therapeutic approaches (Gregory et al. 2008; Snowdon et al. 2011; Seo et al. 2017a; Kozak et al. 2020).

### Conventional anti-cancer therapies regulating SESNs

Several lines of evidence have strongly correlated aberrant miRNAs expression and their target genes to the response to radiotherapy in different types of cancer. Complementary to these results, Cortez et al. (2014) revealed that the radiation sensitivity of lung cancer cells, both in an in vivo and in vitro model, can be altered by upregulating the expression of miR-200c and miR-200a. Their experiments revealed that overexpression of miR-200c increased cellular radiosensitivity by regulating the oxidative stress response genes peroxiredoxin 2 (*PRDX2*), *SESN1* and *Nrf2* (Cortez et al. 2014) directly. Thus, the results support the hypothesis that miR-200c, in response to stress, utilizes the mechanisms that govern intracellular ROS signalling in such a way to make it possible to inhibit DNA double-strand breaks repair, increase the levels of ROS and upregulate p21 (Cortez et al. 2014).

In line with the function of SESNs as stress-inducible proteins that regulate the level of ROS, the functional study revealed an overexpression of SESN2 under radiation-induced oxidative stress in a breast cancer MCF7 cell line. The authors of the study hypothesized that SESN2 blocks ionizing radiation (IR)-induced Akt–mTOR signalling and acts as a radiation sensitizer in breast cancer cells. Moreover, they have shown that SESN2 mediates IR-induced AMPK expression and facilitates the radiosensitization of breast cancer cells (Sanli et al. 2012).

In contrast to the previous studies, Gonzalez et al. (2018) observed that the expression of SESN3 was significantly downregulated during radiotherapy, whereas the expressions of SESN1 and SESN2 remained unchanged in a sample of 26 men with nonmetastatic prostate cancer. Interestingly, the authors hypothesized that oxidative stress induced by radiotherapy may play an important role in individual fatigue experience that could be mediated by downregulation of SESN3. Moreover, they suggested that fatigue reported by patients is connected with SESN proteins implication in regulation of metabolic pathways, oxidative stress and the musculoskeletal system through the mTOR and AMPK signalling pathways (Gonzalez et al. 2018). This intriguing conclusion requires further validation to confirm and explore how SESN3 downregulation influences the role of mitochondrial dysfunction and oxidative stress in radiotherapy-related fatigue.

Another study looked at SESNs’ modulation by chemotherapeutic drugs. Supporting this concept, Kosaka et al. (2017) focused on the unique role of cabazitaxel to induce ROS production in castration-resistant prostate cancer (CRPC) cell lines. It was shown that cabazitaxel exerted higher cytotoxic effects as compared to other taxanes, such as docetaxel and paclitaxel. The authors suggested that such activity of cabazitaxel results from the inhibition of SESN3 expression that leads to increased ROS generation in CRPC cell lines and in an in vivo model. These observation support the concepts that SESN3 prevents the accumulation of ROS and oxidative DNA damage, and increasing ROS generation can make cancerous cells sensitive to chemotherapeutic interventions or other therapeutics that act by further augmenting the production of ROS (Conklin 2004; Fiaschi and Chiarugi 2012; Harris et al. 2015).

In another study, Seo and colleagues have shown the interaction between 5-fluorouracil (5-FU), a chemotherapeutic agent widely used in the treatment of colorectal cancer, and SESN2 expression. The authors clearly demonstrated that colon cancer HCT116 and HT29 cell lines treated with 5-FU expressed a higher level of SESN2 and increased transcripts of SESN1, but not of SESN3 protein. In contrast to the previous study, 5-FU did not induce the ROS production in colon cancer cell lines; thus, a different molecular mechanism was implicated in SESN2 upregulaion. The authors proposed that 5-FU induces the expression of SESN2 in p53-dependent manner (Seo et al. 2017b). The major mechanism of 5-FU activity is related to the inhibition of nucleotide synthase, thereby inducing apoptosis. Interestingly, an additional molecular mechanism underlying the anti-cancer activity of 5-FU is connected with mTOR inhibition (Tomioka et al. 2012). Thus, further investigation into 5-FU anti-cancer activity combining mTOR and SESN protein interactions seems to be of great importance.

An increased interest is observed towards endoplasmic reticulum (ER) stress-inducing drugs such as nelfinavir, bortezomib and HSP90 inhibitors for the treatment of cancer. The observed interest is due to a pro-apoptotic feature of ER stress and the induction of autophagy (Benbrook and Long 2012; Suh et al. 2012). At the molecular level, the treatment of MDA-MB-453 breast cancer cell line, OVCAR3 ovarian cancer cell line and HeLa cervical adenocarcinoma cell line with nelfinavir and the proteasome inhibitor bortezomib resulted in the upregulation of SESN2 protein. Moreover, the cancer cells treated with nelfinavir showed reduced mTOR activity and increased ATF4-mediated SESN2 expression level. Therefore, nelfinavir can act as an effective activator of mTOR inhibitor SESN2. These results link ER stress, mTOR inhibition and autophagy (Brüning et al. 2013). The activity of nelfinavir towards SESN2 upregulation is mediated by ATF4, a transcription factor that activates genes that predominantly control autophagy, protein folding, amino acid metabolism, redox balance and apoptosis (Zielke et al. 2021). Interestingly, cancer cells first and primarily respond to nelfinavir and bortezomib with the upregulation of SESN2, the cell survival mechanism that could lead to alleviation of the oxidative stress. However, prolonged nelfinavir treatment resulted in persistent ER stress that could overcome the anti-oxidative mechanisms and trigger the autophagy and apoptotic cell death. The authors speculated that constant upregulation of SESN2 in response to ER- stress leads to direct inhibition of mTOR and autophagy- promoting mechanism (Brüning et al. 2013). Therefore, it is worth stressing that exposition time for chemotherapeutic is important condition for fully developing their ani-cancerous effect.

In the field of cancer research, accumulating evidence has reported anti-cancer activities of histone deacetylase inhibitors (HDACIs) such as suberoylanilide hydroxamic acid (SAHA), trichostatin A (TSA), and depsipeptide. They are a class of compounds that interfere with the function of histone deacetylase and induce cell cycle arrest, differentiation and apoptosis (Emanuele 1992; Rosato et al. 2003). The main anti-cancer activity of HDACIs is believed to be via an induction of apoptotic cell death in a variety of cancer cells (Duvic et al. 2007). Moreover, it was reported that HDACIs, such as SAHA and TSA, are able to induce autophagy in human cancer cells, an effect related to their anti-cancer property (Liu et al. 2010). The importance of HDACIs in autophagy induction was demonstrated by Zhang et al. (2015), who showed that trichostatin A (TSA) increases the expression of FOXO1 together with nuclear accumulation and enhancement of transcriptional activity. The increased transcriptional activity of FOXO1 leads to increased expression of SESN3 and the inhibition of mTOR signalling pathway. Finally, this interaction loop of TSA–FOXO1–SESN3–mTOR promotes autophagy; however, these pieces of data indicate that autophagy serves as a cell survival mechanism in TSA-treated HCT116 colon cancer cell line and HepG2 hepatoma cell line (Zhang et al. 2015). The authors provide an important insight into the dual role of autophagy. On the one hand, autophagy facilitates cancer cells to tolerate stress induced by a hypoxic microenvironment, starvation and anti-cancer therapies. On the other hand, autophagy plays an important role in the reduction of cytotoxicity of anti-cancer drugs by maintaining energy homeostasis, clearance of ROS or clearing away damaged proteins and organelles (Zhang et al. 2015; Hua et al. 2018). Therefore, it seems reasonable to put some effort into further research to find the key chemotherapy response predictor that could change the direction of the autophagy mechanism from pro-survival towards cancer cell death inducing.

## Conclusions

In our review, we have discussed various aspects of the interplay between anti-cancer substances and SESN proteins’ abnormal expression and how cancer cells respond to these interactions. The presented studies revealed that multiple cancer cells differ with respect to a complex interaction network of SESN proteins implicated in anti-cancer therapy. A detailed insight into the oxidative stress–autophagy–SESNs network is of importance for the application of SESN-modulating anti-cancer drugs in the future. Anti-cancer drugs that manipulate the level of ROS, and in this way influence oxidative stress and autophagy-induced cellular signalling pathways, may be used alone or in combination with other biological response modifiers such as SESN proteins. SESN proteins may be the determinants of sensitivity to anti-cancer treatments as they are activated in response to radiotherapy and many chemotherapeutic drugs and may support cell death in response to certain treatment conditions. The proposed mechanism could be that anti-cancer drugs or radiotherapy administration causes a dysregulation of intracellular signalling pathways and increases oxidative stress. It is important to bear in mind that cancerous cells may try to adapt to a new oxidative stress condition and are capable of dealing with cellular ROS by rapid induction of survival mechanisms promoting carcinogenesis. Given the ability of oxidative stress to activate the SESN proteins, they could be beneficial for tumour cell survival at an early stage of anti-cancer therapy. The mechanisms, such as attenuation of oxidative stress and augmentation of autophagy, which SESN proteins could implicate to promote tumour growth under stress conditions triggered by chemotherapy and radiotherapy, are relatively complex. However, the persistent and severe oxidative stress elicited by proper doses and regime of anti-cancer therapy could overcome the cancerous defence mechanisms at least until new mutations and genetic adaptation occur, which gives a considerable advantage to the new cancer cell population. For this purpose, future preclinical and clinical studies are needed to fully clarify the clinical utility of SESN proteins.

Another important issue is to understand which factors determine whether SESN proteins work as anti-cancerous or pro-cancerous agent in complex process of carcinogenesis. Moreover, it remains controversial and needs further explanation why the expression of SESN proteins is inconsistent in different tumours. Comprehensive studies have shown that low expression of SESN proteins is characteristic of aggressive and primary invasive lung cancer, bladder cancer, colon cancer and prostate cancer. The suggested explanation could be that cancer cells have to silence the antioxidant defence system to sustain proper ROS level which further activates the EMT process and allows for cancer progression. Moreover, the low expression of SESNs in cancer cells could be the consequence of the coexistence of a mutated p53 protein, which regulates their expression. Finally, the inhibition of SESN protein activity could be one of the mechanisms that allows to overstimulate mTORC1 signalling pathways and accelerate the cancer cell proliferation in unfavourable conditions such as nutrient deficiency or ATP depletion. Nevertheless, the controversial issue is why aggressive and metastatic endometrial cancer is characterized by an increased expression of SESN proteins. Bearing in mind the complex and insidious nature of carcinogenesis, the answer to this challenging question could bring some dilemma. One of the proposed explanations could be that higher SESN proteins are a compensatory mechanism and/or adaptive response to specific circumstances of endometrial cancer. However, it requires further validation to fully understand the detailed mechanism responsible for such phenomenon.

In conclusion, SESN proteins seem to deserve the researchers’ attention due to their potential to uncover interesting facts about carcinogenesis and anti-cancer therapy. Their pro-survival role in cancer cells could be used as one of the strategies to nullify the pathways controlled by SESN proteins to alleviate cancer progression. Additionally, developing new comprehensive strategies for the treatment of a particular type of cancer based on the levels of SESN proteins’ expression at different stages of cancer development and progression would be an interesting challenge.

This study is primarily based on the review of the experiments that show SESN proteins as potential modifiers of cancer cells’ response to anti-cancer therapy, but not as direct molecular targets of the applied anti-cancer therapy. We speculate that the observed phenomenon could be attributed to the emerging interest in SESN proteins and a very early stage of research over the SESN proteins’ role in cancer biology. However, diverse functions of SESN proteins, cross talk with a plethora of signalling pathways in a specific cellular context and the complex nature of cancer create only a few obstacles to overcome before SESNs become an effective target of anti-cancer therapy.
